# From Dynamic Live Cell Imaging to 3D Ultrastructure: Novel Integrated Methods for High Pressure Freezing and Correlative Light-Electron Microscopy

**DOI:** 10.1371/journal.pone.0009014

**Published:** 2010-02-03

**Authors:** Coralie Spiegelhalter, Valérie Tosch, Didier Hentsch, Marc Koch, Pascal Kessler, Yannick Schwab, Jocelyn Laporte

**Affiliations:** 1 Imaging Centre, IGBMC (Institut de Génétique et de Biologie Moléculaire et Cellulaire), Illkirch, France; 2 Department of Neurobiology and Genetics, IGBMC, Illkirch, France; 3 Inserm, U964, Illkirch, France; 4 CNRS, UMR7104, Illkirch, France; 5 Université de Strasbourg, Illkirch, France; 6 Collège de France, Chaire de Génétique Humaine, Illkirch, France; University of Nebraska Medical Center, United States of America

## Abstract

**Background:**

In cell biology, the study of proteins and organelles requires the combination of different imaging approaches, from live recordings with light microscopy (LM) to electron microscopy (EM).

**Methodology:**

To correlate dynamic events in adherent cells with both ultrastructural and 3D information, we developed a method for cultured cells that combines confocal time-lapse images of GFP-tagged proteins with electron microscopy. With laser micro-patterned culture substrate, we created coordinates that were conserved at every step of the sample preparation and visualization processes. Specifically designed for cryo-fixation, this method allowed a fast freezing of dynamic events within seconds and their ultrastructural characterization. We provide examples of the dynamic oligomerization of GFP-tagged myotubularin (MTM1) phosphoinositides phosphatase induced by osmotic stress, and of the ultrastructure of membrane tubules dependent on amphiphysin 2 (BIN1) expression.

**Conclusion:**

Accessible and versatile, we show that this approach is efficient to routinely correlate functional and dynamic LM with high resolution morphology by EM, with immuno-EM labeling, with 3D reconstruction using serial immuno-EM or tomography, and with scanning-EM.

## Introduction

Correlative light and electron microscopy (CLEM) defines a variety of techniques that are aimed to visualize the same object with both light microscopy (LM) and electron microscopy (EM). This general description applies to a considerable number of applications where almost all combinations of LM (phase-contrast or fluorescence on living or fixed samples) and EM (immuno-EM, scanning EM, transmission EM, cryo-EM, tomography) have been used during the last few decades [1]. The use of immunofluorescence to select regions of interest (ROI) to be studied by immuno-EM have brought numerous results both on thawed cryosections and on resin sections [2], [3], [4]. In parallel, several successful CLEM experiments have been made on dynamic phenomena in living cells either by recording the phase contrast [5], [6], or by imaging fluorescent dyes [7], [8], [9], [10] and genetically engineered fluorescent proteins [11], [12], [13], [14]. At the EM level, the same objects were then affinity labeled and revealed with electron-dense precipitate [7], [9], [11], [15] or, more commonly used, with gold particles [13], [14]. In rare cases when it is impossible to perform any labeling in EM, as for cryo-EM experiments, the fluorescent signal was used to localize the ROI that is then acquired with high resolution EM [10], [12].

The method to immobilize the specimen has to be as conservative as possible with regard to the cellular ultrastructure and to the antigenicity. Two options have been used to date: chemical fixation and cryofixation. The first usually introduces a compromise between ultrastructure and labeling efficiency, whereas the second is difficult to achieve on thick or highly aqueous samples. The most powerful technique for immuno-detection on chemically fixed samples is probably the one introduced by Tokuyasu [16], [17] as it allows good labeling efficiency with a fine ultrastructural preservation, especially concerning membranes and organelles. This technique has been used with CLEM on cell monolayer [13]. Even though very powerful, cryosections of cell monolayers are technically demanding and probably not suitable for non specialized EM laboratories. Also, the chemical fixation can create artifacts [18], [19] and may not allow for the detection of cytosoluble proteins. High pressure freezing (HPF) followed by freeze substitution and resin embedding is a good alternative [19], [20] as it permits a better preservation of cytoplasmic proteins. Ultramicrotomy at room temperature is also much easier and probably more adapted to routine serial sectioning for immuno-EM and for EM tomography. When using HPF as a fixation method, the choice of the culture substrate is important. Aluminum planchettes [21], microscope grids [18], cellulose capillary tubes [20], [22], sapphire discs [14], [23] and aclar discs [24] have been used with satisfactory results for a large panel of cell types.

A crucial point to succeed in CLEM is to locate at the EM level what has been recorded live in LM. Several strategies have been developed, each of them being adapted to the type of cells to be studied, to the choice of fixation and to the specific EM application used. Growing the cells directly on EM grids has been very successful, especially for cryo-EM [10], [12]. While reaching a high degree of correlation and an excellent ultrastructure, this technique is nevertheless limited to very thin samples, i.e. cell processes, and can hardly be adapted to the cell body or nucleus. Also, no immuno-EM can be performed on frozen samples. Cells have also been grown on culture dishes with landmarks [6], [7], [25] or on marked formvar films [5]. In these cases, chemical fixation was a prerequisite and labeling for EM was either done before embedding [7], [26] or necessitated treatments destructive to cellular integrity [6]. Colombelli and coworkers [27] have introduced a way to define and mark a ROI during the live acquisition of cells grown on glass coverslips. After a necessary chemical fixation, patterns were carved on the glass with a pulsed laser, which allowed a precise selection of the ROI during the ultramicrotomy phase. To avoid chemical fixation, Verkade has developed tools to introduce coordinates on adherent cultured cells in experiments using HPF [14]. The cells were grown on sapphire discs and the coordinates were subsequently given by a metallic finder grid placed on top. Very powerful, this technique has reduced the delay between LM acquisition and fixation for EM to about 5 seconds, which is probably the best time resolved CLEM experiment achieved so far. However, the finder grid used to localize the cells is hard to handle and can lead to cell damage. Furthermore the coordinates are not conserved in the thin sections.

In need of an accessible, versatile but accurate method to perform CLEM on living cells, we developed a technique that combines (i) high resolution live recordings of dynamic phenomena, (ii) fast fixation by HPF for optimal ultrastructure and antigenicity preservation, (iii) precise space coordinates for ROI selection at the EM level, and iv) accessibility to different EM applications. This method uses laser pre-micro-patterned aclar discs as culture substrates, that are compatible with cryofixation, followed by resin embedding. The coordinates of the ROIs are conserved throughout the whole preparation steps, and are still visible on the first sections observed in EM. We were able to record at precise time points GFP-tagged myotubularin oligomerization in osmotically challenged living cells. Furthermore, the micro-patterned cell culture substrate is compatible with time-lapse recordings of long lasting events such as cell migration. With a straight forward re-localization of the ROI at the EM level, serial immuno-EM and EM tomography of membrane tubules induced by amphiphysin 2 overexpression was successfully performed on cells previously acquired by live LM. This working protocol was also applied to correlate LM with scanning electron microscopy.

## Results

### Development of the Culture Substrate with Embedded Coordinates

**Figure 1:**
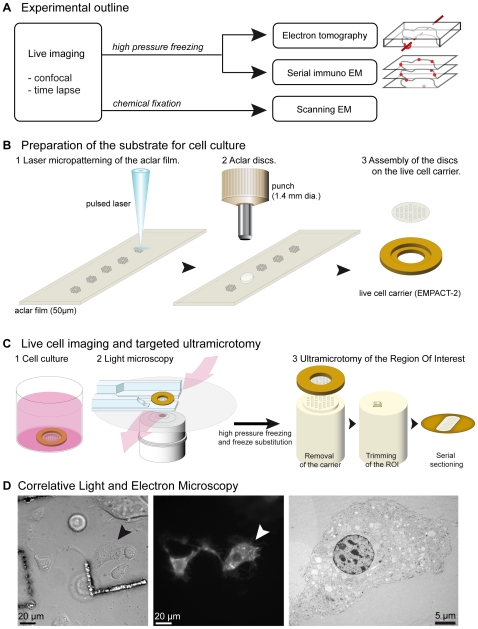
Integrated methods for CLEM applications. (A) Outline of the correlative light-electron microscopy (CLEM) methods on living cells. The micro-patterned culture substrates are useful to perform different types of CLEM, combining confocal microscopy with immuno-labeling on serial sections (transmission-EM), with EM tomography and with scanning-EM. (B) The culture substrates are prepared from aclar films [24] (1) on which a reference grid has been micro-patterned with a laser microdissection microscope. The patterns are cut as 1.4 mm discs with a punch (2) and mounted onto gold plated live cell carriers (3). (C) Cells are seeded on the montage (1) and cultured under normal conditions. For LM recording, the montage is installed on the rapid loader of the EMPACT-2 and placed on the adapted stage of an inverted microscope, allowing continuous perfusion or replacement of the medium (2). After high pressure freezing, freeze substitution and embedding, the carrier is removed from the block (3), leaving prints of the reference coordinates at the block face. Trimming is performed around the region containing the cell of interest that is cut serial and collected on EM grids. (D) COS-1 cells expressing GFP-MTM1 migrated for 7.5 h on the pre-patterned aclar grid coated with collagen, were fixed by high pressure freezing and processed as described above. Coordinates from the grid are still on the first sections, facilitating the retrieval of the previously visualized cell. Laminin and poly-L-lysin coating were successfully tested (not shown). From left to right: bright field, fluorescence and electron microscopy. Examples of different time points and the video are shown online (**Fig. S2 and Video S1**, online).

To handle living cells for HPF and precisely locate regions of interest, pre-patterning of the culture substrate was performed with a laser microdissection microscope directly on aclar films (**Fig. 1B** and **Fig. S1** online). This coordinate referencing does not require the use of an EM grid on top of the culture substrate nor the need to carve landmarks after fixation [14], [27]. The substrate was coated with collagen and cells seeded and cultured under normal conditions (**Fig. 1C-1**). To monitor dynamic events and challenge the cell physiology, cells were maintained, treated and imaged on an inverted confocal microscope, directly on the rapid loader of the high pressure freezing machine (EMPACT-2, Leica Microsystems) which was placed on a tailored perfusion chamber (**Fig. 1C** **-** **2**). Adherent cells attached and behaved normally on this substrate and were able to migrate (**Fig. 1D** **, Video S1** and **Fig. S2** online). As the carved surface of the culture substrate might modify the cells adhesion, we selected cells that were not growing directly on the patterns. For each acquisition, the exact position of the cell of interest was stored relatively to the reference coordinates (**Fig. 1D**, **Fig. S1A-E**). The loader was removed from the confocal set-up and transferred to the EMPACT-2 for HPF. The delay between the last fluorescent acquisition and the complete freezing was around 8 seconds. This time included the removal of the loader from the light microscope, the immersion of the montage into a cryoprotectant consisting of 20% BSA, and the loading process into the HPF apparatus. Each of these steps took 2 to 3 seconds. This relatively fast process allowed ultrastructural imaging of a precise time-point of interest. Freeze substitution and resin embedding of the samples were compatible for sectioning at room temperature and classical EM treatment. When removing the culture substrates from the polymerized resin blocks, the prints of the reference pattern were clearly visible and allowed for precise trimming targeted to the region where the LM acquisitions were performed (**Fig. 1C** **-** [Fig pone-0009014-g002] **3**). Laser micropatterning of the aclar created both positive and negative marks (**Fig. S1G-H**). As a result, the coordinates were visible on the first 10 to 20 thin sections (**Fig. S1I**) thus allowing a straight-forward localization of the cell of interest.

### Correlative Light-Electron Microscopy on Dynamic Phenomena

**Figure 2 pone-0009014-g002:**
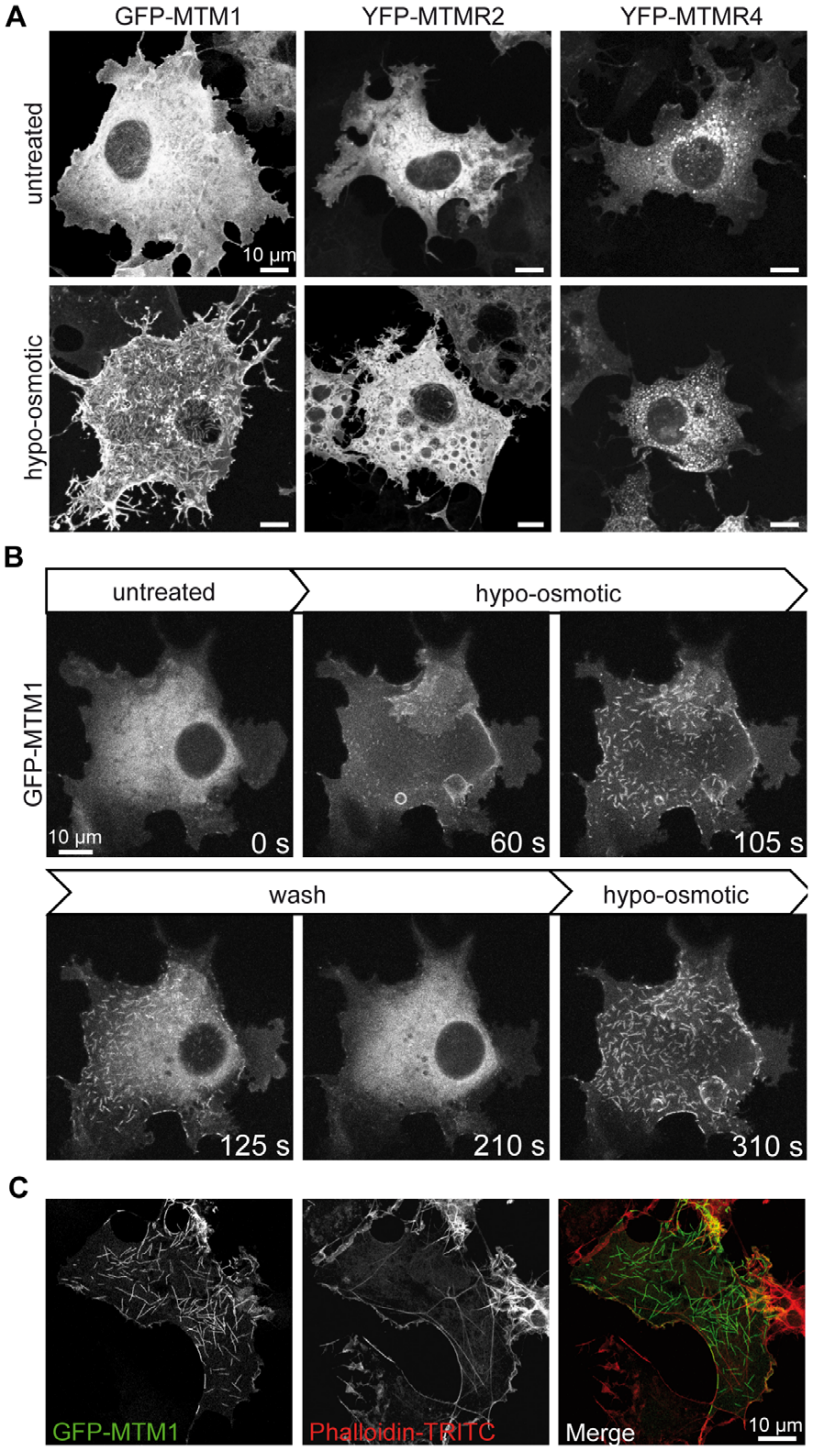
Dynamic needle-like structure formation. (A) COS-1 cells were transfected with GFP or YFP-tagged wild-type myotubularin (MTM1) or close homologous proteins MTMR2 and MTMR4, either untreated or switched to a hypo-osmotic medium for 10 min, and imaged by confocal microscopy. Treatment does not change the subcellular localization of MTMR4 (right panel) and several other YFP-tagged proteins tested (not shown), while it promotes recruitment of MTMR2 (middle panel) to big vacuoles induced by the treatment, and the formation of needle-like structures by GFP-MTM1 (left panel). (B) The formation of needles is reversible for at least 3 times (two medium switches shown here). (C) The needles do not colocalize with actin (labeled with phalloidin-Texas Red).

To exemplify the accessibility of the method, we characterized the oligomerization of GFP-tagged myotubularin induced by hypo-osmotic treatment at the ultrastructural level. Myotubularin (MTM1, NM_000252) is a 3-phosphoinositides phosphatase mutated in a severe form of congenital myopathies called X-linked centronuclear myopathy or myotubular myopathy [28]. It dephosphorylates the phosphatidylinositol 3-phosphate (PtdIns3*P*) and PtdIns(3,5)*P*_2_ and is believed to be implicated in membrane remodeling and transport. We noted that GFP-MTM1 localizes in the cytosol and at the plasma membrane under normal conditions, and forms “needle-like” structures upon hypo-osmotic treatment, a condition that increases the level of PtdIns(3,5)*P*_2_ (**Fig. 2A** **, Fig. S3** online) [29]. This nucleation is enhanced by the GFP tag, as untagged proteins usually retain normal localization, and is dependent on the myotubularin sequence as the localization of close homologues is not similarly affected (**Fig. 2A** **, Fig. S4** online, and Berger *et al.* [30]). The needles, that start to be visible after 30 seconds of treatment, stop elongating after 5 minutes. This process can be reversed several times by switching media (**Fig. 2B**, **Video S2 and S3** online). As these needles do not colocalize with the actin cytoskeleton (**Fig. 2C**), establishment of a high-throughput CLEM protocol was necessary to retrieve and analyze their ultrastructure. This phenomenon is highly dynamic and the water-enriched hypo-osmotic medium provided additional challenges for the set-up of CLEM.

**Figure 3:**
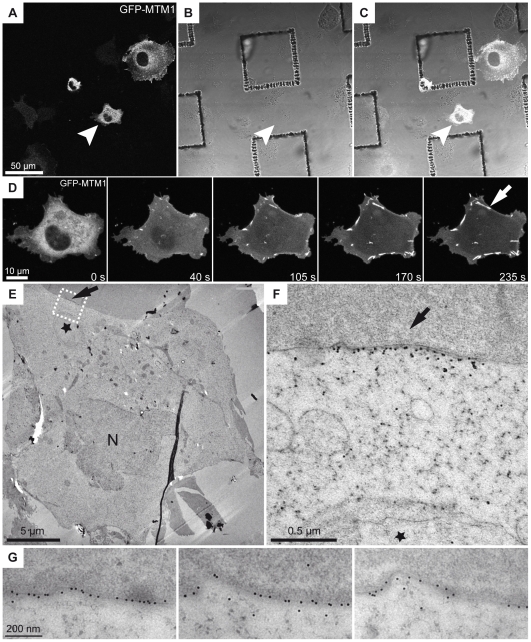
Correlative light and electron microscopy of the needle-like structures. COS-1 cells transfected with GFP-tagged MTM1 and plated onto collagen coated pre-patterned aclar grids were treated with hypo-osmotic shock, imaged by time-lapse confocal microscopy then fixed by high pressure freezing. (A–C) The reference coordinates are used to record the position of the selected cell with fluorescence (A), bright field microscopy (B) or both (C). (D) Representative images of the time-lapse video (Supplementary video 4, online), the image at 235 s was the last image before high pressure freezing. (E–F) Examples of a needle structure in immuno-EM labeling using anti-GFP antibody. Arrows in (D–F) point to the same structure. Needles are found associated with the plasma membrane. The cell position, its global shape, the position of the nucleus (N) and of a large vacuole (star) were used to confirm the identity of the cell and to perform the correlation. (G) Micrographs of consecutive sections from the same region as in F showing the distribution of the gold labeling (anti-GFP) at the plasma membrane.

We performed CLEM at different time-points between 1.5 and 10 min; an example at 4 min during needle formation is shown in **Figure 3** (see also **Video S4)**. The structures observed by transmission-EM after anti-GFP immunogold labeling were easily fitted with the last fluorescent image acquired just before HPF, based on the reference grid. Needles appear systematically located at the plasma membrane (**Fig. 3F and 3G** **; Fig. S5**). They do not correspond to membrane tubules, as hypothesized from the known function of myotubularin, but represent most likely oligomers, that were previously described *in vitro* after mixing recombinant myotubularin with phosphoinositides [31].

Overall, this protocol allows easy culture and treatment of adherent cells on a substrate compatible with CLEM and is applicable to the study of dynamic phenomena.

### Correlative Light-Scanning Microscopy

**Figure 4:**
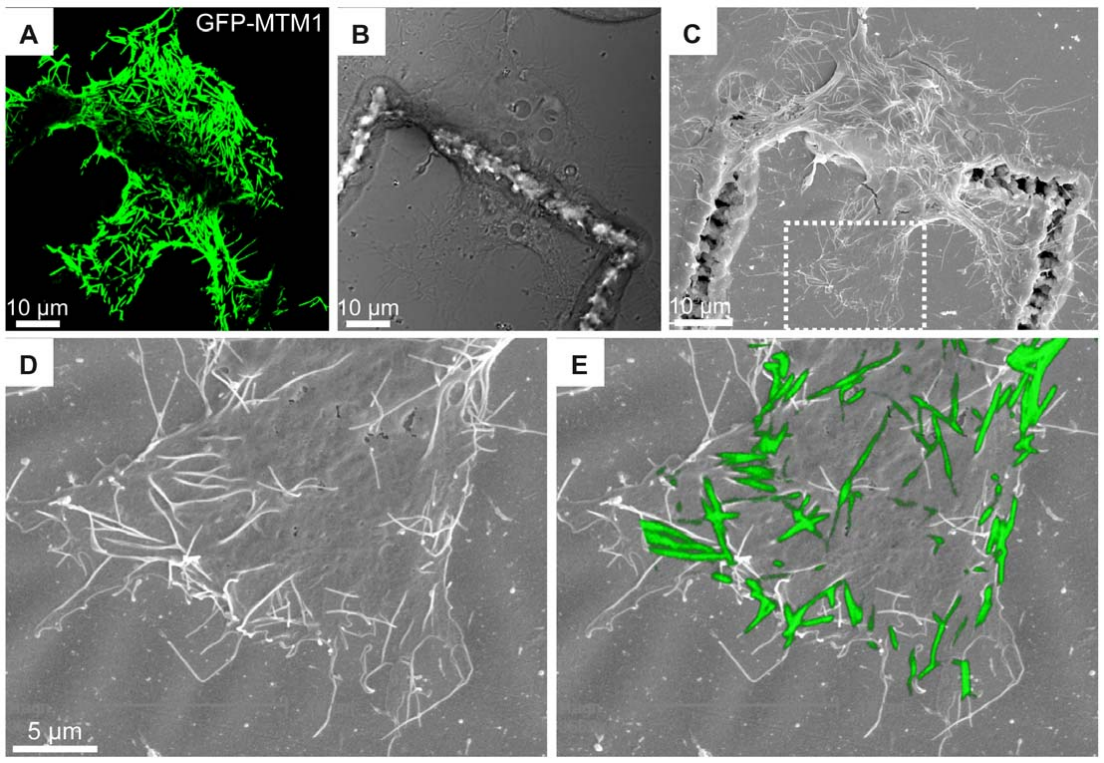
Correlative light and scanning electron microscopy. COS-1 cells transfected with GFP-tagged MTM1 were treated with a hypo-osmotic shock for 10 min, imaged with confocal microscopy and processed for scanning-EM. (A) Confocal fluorescence, (B) bright field and (C) scanning-EM images of a GFP-positive cell on top of the reference grid. (D) Enlargement of (C). (E) The superposition of confocal (z-stack of 3.52 µm) and scanning-EM images shows that the needles do not fit with filopodia but correspond to dorsal ruffles.

As the reference grid is also visible by scanning-EM, we performed correlative microscopy on GFP-MTM1 positive cells after 10 min of hypo-osmotic treatment. Immediately after fluorescence imaging, cells were chemically fixed and processed for conventional scanning electron microscopy (SEM). The GFP-positive signal acquired before fixation was fitted with the scanning-EM picture and corresponds mainly to contrasted structures on top of the cell (**Fig. 4**). Some distortions were noted in the SEM images compared to the fluorescence, and are probably due to shrinking of the cell during dehydration. Needles do not extensively fit with filopodia extending onto the substratum but likely represent cytosolic structures beneath the plasma membrane, that appear contrasted due to the dehydration process. These structures were estimated to range from 1.5 to 5 µm in length.

Combining immuno-EM and scanning-EM allowed us to discriminate between filopodia-like structures extending outside the cell and cytosolic structures beneath the plasma membrane. Correlation with scanning-EM should more generally be applicable to the study of plasma membrane and cell shape remodeling during cell migration, upon desired treatment and time period.

### 3D Reconstruction with Correlative Light-Electron Tomography and Serial Immuno-EM

**Figure 5:**
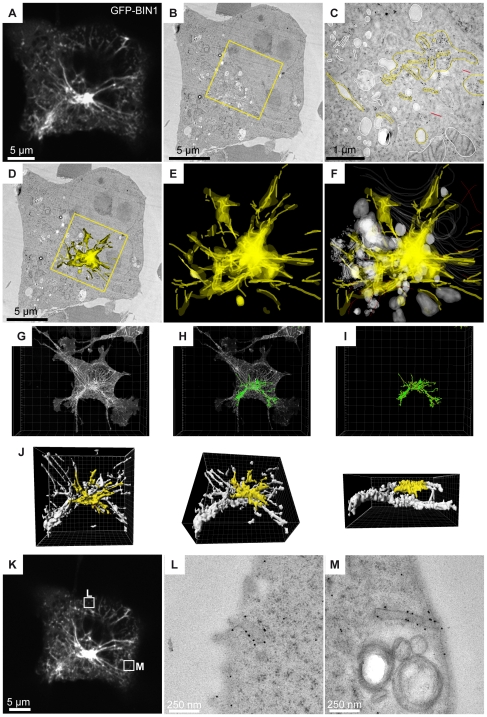
CLEM and serial immuno-EM. COS-1 cells transfected with GFP-tagged BIN1 were imaged by confocal microscopy, fixed by high pressure freezing and processed for 60 nm thick serial sections. Sections were then labeled with anti-GFP antibody and gold particles. (A) Representative image (z projection of 3 confocal sections of 0.28 µm in depth) of the fluorescent BIN1 tubules radiating from/to the perinuclear region. (B) One over 30 serial thin sections showing the transmission-EM image of the same cell as in (A). (C) At higher magnification, the immunostaining is visible over various perinuclear structures highlighted in yellow. Other organelles such as mitochondria, Golgi complex, endosomal vesicles, that were unstained, are modeled in white. (D) When rendering the whole stack of serial sections, the immunogold labeling can be projected onto the representative transmission-EM picture, showing a good correlation with the dense perinuclear GFP signal recorded on living cells. (E and F) The model displays the distribution of the anti GFP immunogold staining in the volume of the perinuclear region, (G–J) The full z stack recording was processed with Imaris to reconstruct a 3D model (green) of the GFP fluorescence. The region analyzed by EM has been color-coded in yellow, showing a good correlation with the gold labeling displayed in D-F. (K–M) Correlation analysis of other z planes showed localization of GFP-BIN1 at plasma membrane invaginations. The confocal image shown in K corresponds to the Z projection of the five stacks shown in the Figure S6 (online). The EM micrograph of the corresponding thin sections as well as additional BIN1-positive membrane tubules and structures are shown in Figure S6 (online).

To further assess the accuracy of our CLEM protocols, we wanted to correlate the 3D information collected from confocal acquisitions, with both serial immuno-EM and EM tomography. For this purpose, we selected a model where an intracellular GFP signal could be associated with the labeling of an identified intracellular structure, such as compartments or organelles. As MTM1 needles were found to be associated to the plasma membrane and not to intracellular structures, this model was not suitable for such approaches. We therefore focused on another protein of the membrane remodeling pathway that is similarly mutated in centronuclear myopathy, BIN1 (also called amphiphysin 2, NM_004305) [32]. BIN1 is also a potential tumor suppressor implicated in endocytosis [33] and induces membrane tubulation upon overexpression in cultured cells (**Fig. 5A**) [32], [34].

Immuno-EM correlated to the fluorescence image identified the BIN1-positive structures as membrane tubules with a variable width ranging from 40 to 90 nm. These membrane tubules appear to radiate from perinuclear regions (**Fig. 5A,B**). The analysis of 30 serial sections not only allowed the identification of the labeled organelles at the subcellular level but also their position relative to other unlabeled structures such as the plasma membrane, the nucleus, the Golgi complex, endosomal vesicles and mitochondria (**Fig. 5C** **; Video S5**). The model built from the serial immuno-EM allowed a clear fitting of the immunogold labeling with the GFP signal previously acquired on living cells (**Fig. 5D–F**). Furthermore, the model based on the gold labeled structures fits the 3D model reconstructed from the confocal acquisition (**Fig. 5G–J** **; Video S6 and S7**) Plasma membrane invaginations were also enriched with GFP-BIN1 (**Fig. 5K–M** **, Fig. S6** online), showing that membrane tubules emanate from the plasma membrane and direct towards the perinuclear region.

**Figure 6:**
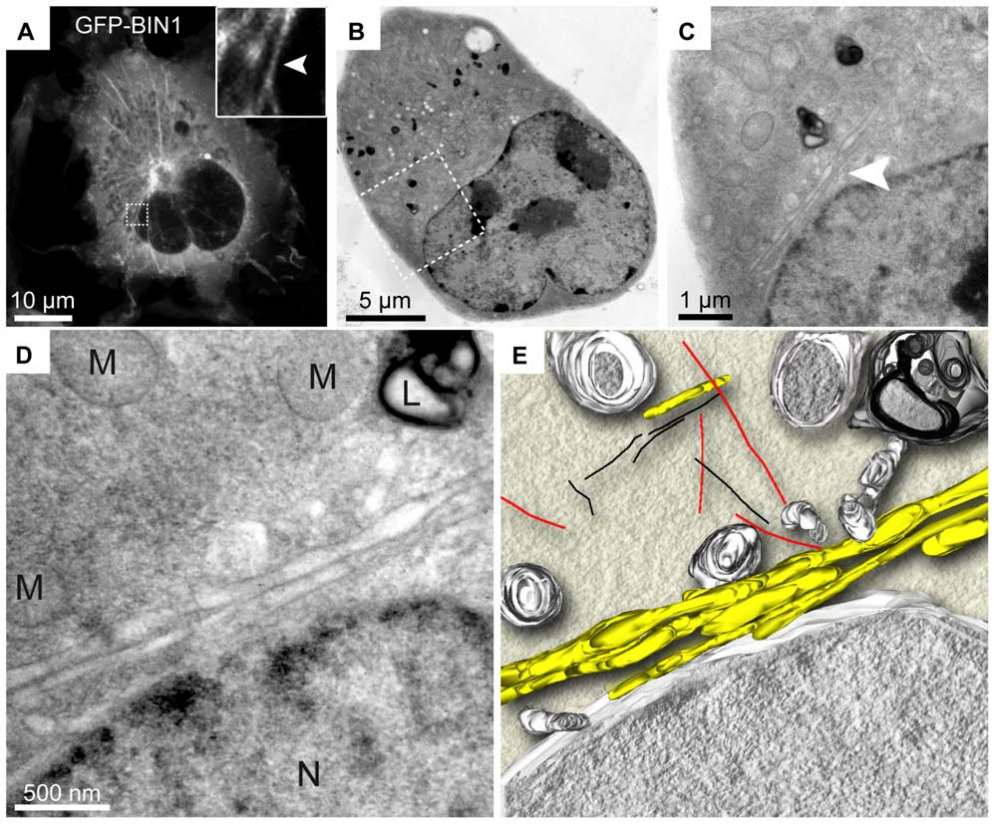
CLEM and tomography reconstruction. COS-1 cells transfected with GFP-BIN1 were imaged by confocal microscopy, fixed by high pressure freezing and processed to 200 nm thick sections. A tomogram was reconstructed from 139 tilted images (from −69 to 69°) and manually segmented to highlight the fluorescent tubules observed previously by confocal microscopy. (A) Representative confocal z-stack projection of a transfected cell, showing GFP-BIN1 tubules. Insert: magnified view of a tubule adjacent to the nucleus. (B) Corresponding transmission-EM picture. (C and D) Higher magnifications of the area selected in (B) showing a bundle of tubules (arrowhead) passing next to the cell nucleus, in the same region highlighted in (A). (E) Top view of the 3D model depicting membrane tubules in yellow, mitochondria, nuclear envelope, endosomal vesicles and lysosomes in grey, microtubules in red and actin filament in black. A 3D video of the tomogram is available (Video S8, online). L: lysosome; M: mitochondria; N: nucleus.

EM tomography and segmentation from 200 nm thick sections were applied to better define the relationship between these membrane tubules and their environment (**Fig. 6**). Volume reconstruction showed that a given membrane tubule can vary in width. Although confocal microscopy shows a single tubular GFP signal (**Fig. 6A**), the corresponding area studied by EM tomography reveals a bundle of several tubules (**Fig. 6B–E**; **Video S8**).

In conclusion, our correlative approach is versatile and can be used to assess membrane tubules localization and conformation in three dimensions both through serial immuno-EM and tomography.

## Discussion

Our interest in CLEM was to link dynamic cell imaging, i.e. fluorescence time-lapse microscopy, with 3D imaging at the ultrastructural level (with scanning-EM, serial immuno-EM and tomography). The available protocols were not suitable to our needs as we wanted a simple technique versatile enough to be applied to various models. We developed a method based on a new culture substrate for adherent cells, in which reference coordinates are included by laser micro-patterning on aclar disc prior to cell seeding. Combined with high pressure freezing to allow optimal ultrastructure and epitope preservation, this method was suitable for CLEM of dynamic phenomena in the range of a few seconds to several hours, enabling imaging at precise time points. In this study, we have chosen to record cells in their culture medium or in an osmotically challenging medium. For the dynamic study of the GFP-MTM1 needles formation, we obtained efficient freezing without cryoprotection (not shown). However, adding a cryoprotective step, when transferring the recorded cells to the high pressure freezing apparatus, gave a better freezing quality by diminishing the occurrence of ice formation and damages. As a consequence, the time spent between the last LM image and cryofixation of cells was longer (8 seconds) than what can be achieved without this step (5 seconds). A new feature compared to previous protocols [14] is the embedding of reference coordinates which are present throughout all sample preparation steps for EM. This coordinate system does not require any handling of the culture substrate prior to the imaging step and is therefore not damaging to the cell culture. Furthermore the cell monolayer is grown on the landmarks, which makes the later visible on the first thin EM sections and allows an easy re-localization of subcellular domains previously acquired from living cells. Compared to existing protocols based on cryosections [13], [35], working on embedded cell monolayers and sectioning them at room temperature make this method applicable on a routine basis.

Light microscopy gives access to the functionality of a given protein in physiological conditions, whereas EM enables precise localization of the same protein with respect to the surrounding anatomical organization of the cell, at a much better resolution. CLEM performed on living cultured cells is of particular interest when a small proportion of a cell population is to be studied, for example transfected cells, or when a heterogeneous cell population is employed, or when different subcellular events are to be characterized within a unique cell.

We applied our method to the study of two proteins implicated in membrane remodeling and mutated in neuromuscular disorders. We were able to obtain information on protein localization in the volume of cells by correlating the oligomerization of GFP-tagged MTM1 with immunogold labeling observed by transmission-EM or with cell surface analysis done by scanning-EM. Dynamic correlative light and scanning EM was also recently used to analyze cytosolic actin structures [6]. The combination of imaging approaches showed that GFP-MTM1 oligomerizes into needle-like structures, a phenomenon most probably enhanced by GFP-tagging and altered phosphoinositide levels induced by osmotic stress. These needles polymerize at the plasma membrane, suggesting the possible involvement of myotubularin in membrane subdomain scaffolding or recognition. Membrane tubules induced by the muscle-specific isoform of BIN1 converge towards the perinuclear region. 3D reconstruction from serial immuno-EM or from EM tomography showed that they do not originate from internal membranes but invaginate from the plasma membrane, suggesting that BIN1 promotes strong curvature from plasma membrane subdomains as anticipated by previous studies [34].

In conclusion, this accessible method provides researchers with the possibility to characterize dynamic events at the ultra-structural level with EM, immuno-EM, tomography and scanning EM. Improvement of this method towards a better resolution in the z direction at the light microscopy and a quicker fixation will require the coupling with recent super-resolutive light microscopy techniques and the development of novel high pressure freezing devices, respectively.

## Materials and Methods

### DNA Constructs and Cell Culture

For eukaryotic cell transfection, human MTM1, MTMR2 (NM_016156) and MTMR4 (NM_004687) cDNA (Laporte *et al.* [28] and Kazusa human cDNA project) and BIN1 muscle-specifc isoform 8 cDNA (a gift from P. De Camilli) were cloned into pEGFP-C1 (Clontech) and overexpressed as an N-terminal EGFP-tagged protein; MTM1 was also cloned into pSG5 hER-B10 and pCMVTag 3B Myc vectors and overexpressed as an N-terminal B10 and Myc-tagged proteins respectively. COS-1 cells were cultured in Dulbecco's modified Eagle's medium (DMEM) supplemented with 5% FCS and 40 mg/l gentamicin in a 5% CO_2_ incubator at 37°C. Cells were grown on 22-mm^2^ cover slips or on aclar discs coated with collagen, and were transfected at 80% confluency with 1.5 µg of DNA constructs using the Fugene-6 reagent, following the manufacturer's instructions (Roche). After 24 h of transfection, cells were hypo-osmotically shocked for 10 min in 25% medium (diluted with water). Hyper-osmotic medium was the normal culture medium supplemented with 0.9M Sorbitol. Cells grown on 22-mm^2^ cover slips for conventional fluorescence microscopy were fixed with 4% paraformaldehyde for 12 h at 4°C. For immunolabelling, cells were subsequently permeabilized in PBS with 0.2% Triton X-100 and washed with PBS. Nonspecific sites were blocked in PBS with 10% FCS and 0.1% Triton X-100. The cellular localization of B10-MTM1 and Myc-MTM1 was assessed by incubation for 1 h with monoclonal B10 and Myc antibodies (IGBMC antibody platform), diluted at 1∶800 and 1∶1000 respectively. After washing with PBS with 0.1% Triton X-100, we detected immunostaining by incubation for 1 h with an anti-mouse antibody-Alexa Fluor 594 (Invitrogen).

### Correlative Light-Electron Microscopy

The culture substrates were assembled before seeding of the cells. They consisted of a laser micro-patterned aclar film mounted on HPF live cell carriers (Leica Microsystems ref: 16707897). Laser micro-patterning was performed with the pulsed laser of the Leica Laser Microdissection microscope (LMD 6000), using the controlling software version 6.5, and consisted of an asymmetric mesh of 70 µm squares (see **Fig. S1** online). The 10X/0,25 P (PL FLUOTARD 556000 Leica Microsystems, Germany) lens objective was used, with specific laser parameters (power: 101 to 104; speed: 6; specimen balance: 0; offset: 60). From 150 to 200 patterns were carved on a rectangular (75*25 mm) 2 mil ( = 50.8 µm) aclar film (E.M.S.). A punching device consisting of a tapped stainless steel tube (Goodfellow external diameter 1.65 mm; internal diameter 1.39 mm; wall thickness 0.13 mm) mounted on a silicon cylindrical handle was used to cut discs (1.4 mm in diameter) from the carved film. The discs were glued with loctite 350 on the HPF carriers (**Fig. S1J** online). The UV light necessary for curing of the glue also sterilized the montages.

Fluorescence was examined with the Leica TCS SP2-AOBS confocal microscope (Argon laser, 488 nm). The stage for the inverted confocal microscope supplied with the HPF machine (EMPACT-2 Leica Microsystems) was not adapted to the use of high magnification lenses (63X, 100X). We therefore built a new stage with a larger central hole (external diameter 43 mm; internal diameter 36 mm). A round coverslip (diameter 42 mm, thickness 0.17 mm, VWR) was installed on the stage and sealed with silicon paste to avoid medium leaking. A water immersion 63X lens objective (63x/1.20W Corr, apo) was used to account for the large working distance introduced by the montage. We used a perfusion device for the renewal or exchange of the culture media, consisting of 2 Teflon tubes (Tygon, Fisher Bioblock SA) and of a peristaltic pump (Ismatec). The liquid flow rate was set to 2 to 3 ml/min and the medium was heated to 37°C.

Time-lapse experiments were performed on an inverted epifluorescence microscope (Leica DM IRE2) equipped with an incubation chamber allowing the control of temperature (37°C) and CO_2_ (5%) (Pecon GmbH, Germany). Up to 12 carriers were placed upside up and recorded sequentially for 7 to 11 hours.

For high pressure freezing and freeze substitution, the visualization of the cultured cell was performed with the live cell carriers mounted on the rapid loader of the EMPACT-2. At chosen time points, the montage was withdrawn from the microscope stage, and transferred to the EMPACT-2 equipped with the RTS extension. For cryoprotection before freezing, the cells were dipped into a solution of 20% BSA in the culture medium. Without cryoprotection, delays of about 5 s between the last image acquisition and the freezing were achieved. With the cryoprotection step, this delay was about 8 s. The images shown here were taken from cryoprotected cells, as the freezing quality was improved. After freezing, the samples were collected in liquid nitrogen until further processing was required. For immunogold analysis, dehydration was performed at −90°C for at least 48 h in pure acetone containing 0.1% uranyl acetate using the EM-AFS freeze substitution unit (Leica Microsystems). Temperature was then raised to −50°C (5°C/h) and after 24 h in the mix, the cells were extensively rinsed first with acetone and then with pure ethanol. Infiltration was performed at −50°C with graded concentrations of lowicryl HM20 over 24 h. Polymerisation was performed with UV light at −50°C for 48 h and at room temperature for 48 h. For morphological experiments, dehydration at −90°C lasted at least 48 h in acetone containing 2% osmium tetroxide, 0.25% uranyl acetate, 0.25% glutaraldehyde and 1% H_2_O. Temperature was raised to −30°C (5°C/h) and cells were left in the mix for 24 h, rinsed with acetone and infiltrated in graded epon/araldite mix. When the resin concentration reached 70%, the temperature was raised to 20°C and the samples were placed in 3 consecutive baths of pure resin (lasting 1 h each) before polymerization at 60°C for 48 h. For polymerization, the carriers were installed in flow-through embedding molds (Leica Microsystems) filled with the resin. After curing, the blocks were removed from the molds and installed in the block holder of the ultramicrotome. The carrier was then removed with a razor blade, by applying a force on the edge, at the interface between the metal and the resin. By doing so, the carrier usually came with the aclar disc, leaving a block face on which the imprints of the micro-patterned coordinates were easily recognizable. The selection of the recorded ROI was performed by trimming small square regions (around 200 µm in width) with a beveled edge diamond knife (cryotrim, Diatome). Thin (50 to 60 nm) and thick sections (200 nm) were collected on formvar-carbon coated copper slot grids for morphological or tomography experiments, and on formvar-carbon coated nickel slot grids for immunogold labeling.

Immunogold labeling was performed on the EM-IGL automate (Leica Microsystems). Rabbit polyclonal anti-GFP (AbCam 6556) was revealed with a 10 nm gold coupled protein A (Utrecht, Netherlands). Grids were contrasted with uranyl acetate and lead citrate before observation with a Philips CM12 transmisison electron microscope operated at 80 kV. Images were acquired with an Orius 1000 CCD camera (Gatan).

For electron tomography, semithin sections were post-stained with uranyl acetate (15 min) and lead citrate (7 min). Ten nm colloidal gold particles were applied on one side of the grid to be used as fiducial markers. Automated data acquisition of the single tilt series through an angular range of −69° to +69° with 1° increments was performed using a field emission gun electron microscope operating at 200 kV (Tecnai F20; FEI Company, Eindhoven, The Netherlands). Images were acquired on a Gatan 2K CCD camera controlled by the Xplore3D software (FEI). Tomograms and 3D models were computed using etomo and Imod [36], [37].

### Scanning Electron Microscopy

Cells were fixed with 2.5% glutaraldehyde in 0.1M cacodylate buffer for 1 h at room temperature followed by a 1 hour post-fixation in 1% osmium tetroxide at 4°C. Dehydration was performed in graded ethanol and hexamethyldisilazane. After palladium gold coating in a Baltec SCD005 sputter coater cells were observed with a Philips XL20 SEM operating at 12 kV.

### Images Treatment and Analysis

Confocal pictures were processed with Tcstk software (Jean-Luc Vonesch, IGBMC) and edited using Dvrtk software (Jean-Luc Vonesch, IGBMC) and Photoshop 7.0 (Adobe) or Fotographix (L. Madhavan). Acquired images were edited and fitted (fluorescence vs EM) using ImageJ and Photoshop (Adobe). The modelization of the confocal acquisition was performed with Imaris (Bitplane).

## Supporting Information

Figure S1 Reference grid imprinted onto the cell culture substrate. (A) Scheme of the reference grid for the laser microdissection microscope. The patterned aclar substrate was imaged under an epifluorescence microscope using excitation filters BP 545/30 (B), BP 480/40 (C) and BP 360/40 (D). The grid fluorescence is much fainter than the GFP signal of tagged proteins. (E) The reference grid is visible in brightfield and by scanning electron microscopy (F). (G-I) Due to the melting of the aclar, positive and negative patterns are imprinted as shown by scanning EM (G). As a result, after polymerization and removal of the culture substrate (H), the pattern appears as negative and positive marks leaving visible holes (I) on the first EM sections. (J) Pictures of the pre-patterned substrate mounted onto gold plated live cell carriers.
(6.83 MB TIF)

Figure S2 Correlative light-electron microscopy on migrating cells. Migration of COS-1 cells expressing GFP-MTM1 on the pre-patterned aclar grid coated with collagen. (A) Brightfield timelapse acquisition. (B) Corresponding fluorescence images; the arrow indicates the cell of interest. (C) A representative EM image of the cell pointed out in (A) and (B). (D) A high magnification image on a different section from the cell in (C).
(6.38 MB TIF)

Figure S3 Details of the needle-like structures formed by GFP-MTM1 upon hypo-osmotic treatment. COS-1 cells were transfected with GFP-tagged MTM1 and treated for 10 min with hyper-osmotic or hypo-osmotic conditions. Protein localization was similar under normal or hyper-osmotic media. (A) xy confocal images showing the needle structures within the cell. (B) z projection image of the cell apex showing the needle organization. (C) xz projection from the image shown in (B) suggesting the presence of the needle structures at the plasma membrane and not inside the cytosol.
(1.44 MB TIF)

Figure S4 The formation of needle-like structures by MTM1 is enhanced by a GFP-tag. COS-1 cells were transfected with N-terminal B10, Myc or GFP-tagged MTM1, either untreated or switched to a hypo-osmotic medium for 10 min, and imaged by confocal microscopy. Upper left image depicts the localization of MTM1 at cell protrusions in highly over-expressing cells, while low over-expressing cells display a more diffuse cytosolic pattern. Most of B10- and Myc-tagged MTM1 transfected cells display a cytosolic pattern although some contained needle-like structures.
(2.56 MB TIF)

Figure S5 Additional GFP-positive needles observed on TEM sections after immuno-EM experiments, related to Figure 3. The gold particles, revealing the accumulation of GFP-MTM1 proteins, are concentrated at sub-domains of the plasma membrane. The inserts show magnified views of the boxed areas.
(5.07 MB TIF)

Figure S6 BIN1-positive structures observed after CLEM and immuno-EM. (A) Consecutive z stacks (0.28 µm thick) of the apex of the cell shown in figure 5. The white arrowheads show the region of the cell corresponding to the EM pictures shown in B-C-D and in figure 5L. The empty arrowheads point to the region where the pictures shown in E-F-G and in figure 5M were taken. (H) gold particles were associated to fine tubules (FT) and to the membrane of enlarged tubules (ET). Internal vesicles of a multivesicular body (MVB) were also stained. (I) Gold labeling was also found of vesicles of various sizes and (J) on more complex and reticulated membrane structures. These structures seem to be induced by the over-expression of the BIN1 construct as that do not appear in non transfected cells. Their identity, i.e. endosomal or lysosomal, post-golgi, is not known.
(6.55 MB TIF)

Video S1 Time-lapse video of GFP-MTM1 positive cells. Cells are migrating on the CLEM substrate coated with collagen, corresponding to Figure 1D. Cells are able to send protrusions and to migrate. Scale bars 20 µm.
(0.61 MB MOV)

Video S2 Time-lapse fluorescence microscopy of needle formation. A ten minutes acquisition of COS-1 cells transfected with GFP-tagged MTM1. The movie starts when the normal culture medium is replaced by the hypo-osmotic medium.
(0.31 MB MOV)

Video S3 Reversibility of needle formation. Time-lapse fluorescence microscopy of needle formation and reversibility in COS-1 cells transfected with GFP-tagged MTM1, corresponding to Figure 3. Normal and hypo-osmotic media were switched as indicated. Scale bars 10 µm.
(5.82 MB MOV)

Video S4 Time-lapse fluorescence microscopy before high pressure freezing. Needle formation in COS-1 cells transfected with GFP-tagged MTM1 before high pressure freezing and processing for correlative cryo-EM, corresponding to Figure 3. Hypo-osmotic medium perfusion started at time 0. Scale bars 10 µm.
(0.37 MB MOV)

Video S5 Modelization of the gold labeled structures. Thirty serial sections have been analyzed to segment the structures that were labeled with gold in yellow. The movie shows the aligned serial sections and the modelized portion of the cell shown in figure 5.
(11.55 MB MOV)

Video S6 Model of the GFP-BIN1 fluorescent signal. The fluorescent signal has been thresholded to modelize the GFP-BIN1 tubules induced by overexpression in COS-1 cells. Rotation of the model, superimposed with the fluorescent signal, shows the organization of the tubular network inside the cell. Snapshots of this movie are shown in figure 5 for correlation with immuno-EM.
(12.15 MB MOV)

Video S7 Model of the GFP-BIN1 localization. The same sequence as in the previous video, but without the fluorescent signal.
(11.57 MB MOV)

Video S8 Subcellular organization on the GFP-BIN1 tubules. 3D representation of the model constructed from the tomogram of the perinuclear region shown in Figure 6.
(8.49 MB MOV)
