# Supplementary figures and images for: From Dynamic Live Cell Imaging to 3D Ultrastructure: Novel Integrated Methods for High Pressure Freezing and Correlative Light-Electron Microscopy
Source: PLoS One. 2010 Feb 3;5(2):e9014. doi: 10.1371/journal.pone.0009014 (PMC2815783; doi:10.1371/journal.pone.0009014)

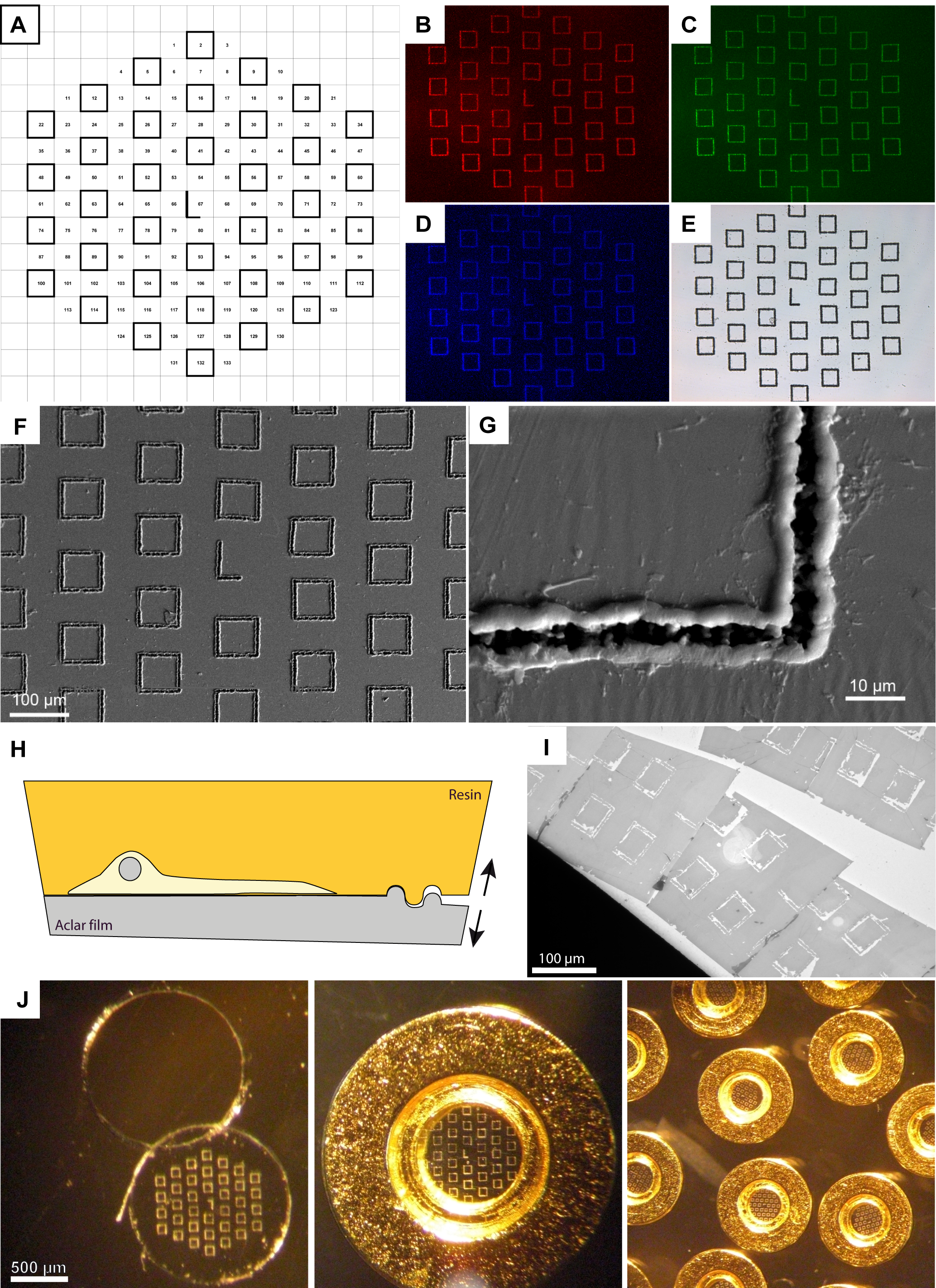

Supplement: Figure S1 — Reference grid imprinted onto the cell culture substrate. (A) Scheme of the reference grid for the laser microdissection microscope. The patterned aclar substrate was imaged under an epifluorescence microscope using excitation filters BP 545/30 (B), BP 480/40 (C) and BP 360/40 (D). The grid fluorescence is much fainter than the GFP signal of tagged proteins. (E) The reference grid is visible in brightfield and by scanning electron microscopy (F). (G-I) Due to the melting of the aclar, positive and negative patterns are imprinted as shown by scanning EM (G). As a result, after polymerization and removal of the culture substrate (H), the pattern appears as negative and positive marks leaving visible holes (I) on the first EM sections. (J) Pictures of the pre-patterned substrate mounted onto gold plated live cell carriers. (6.83 MB TIF) [file pone.0009014.s001.tif]

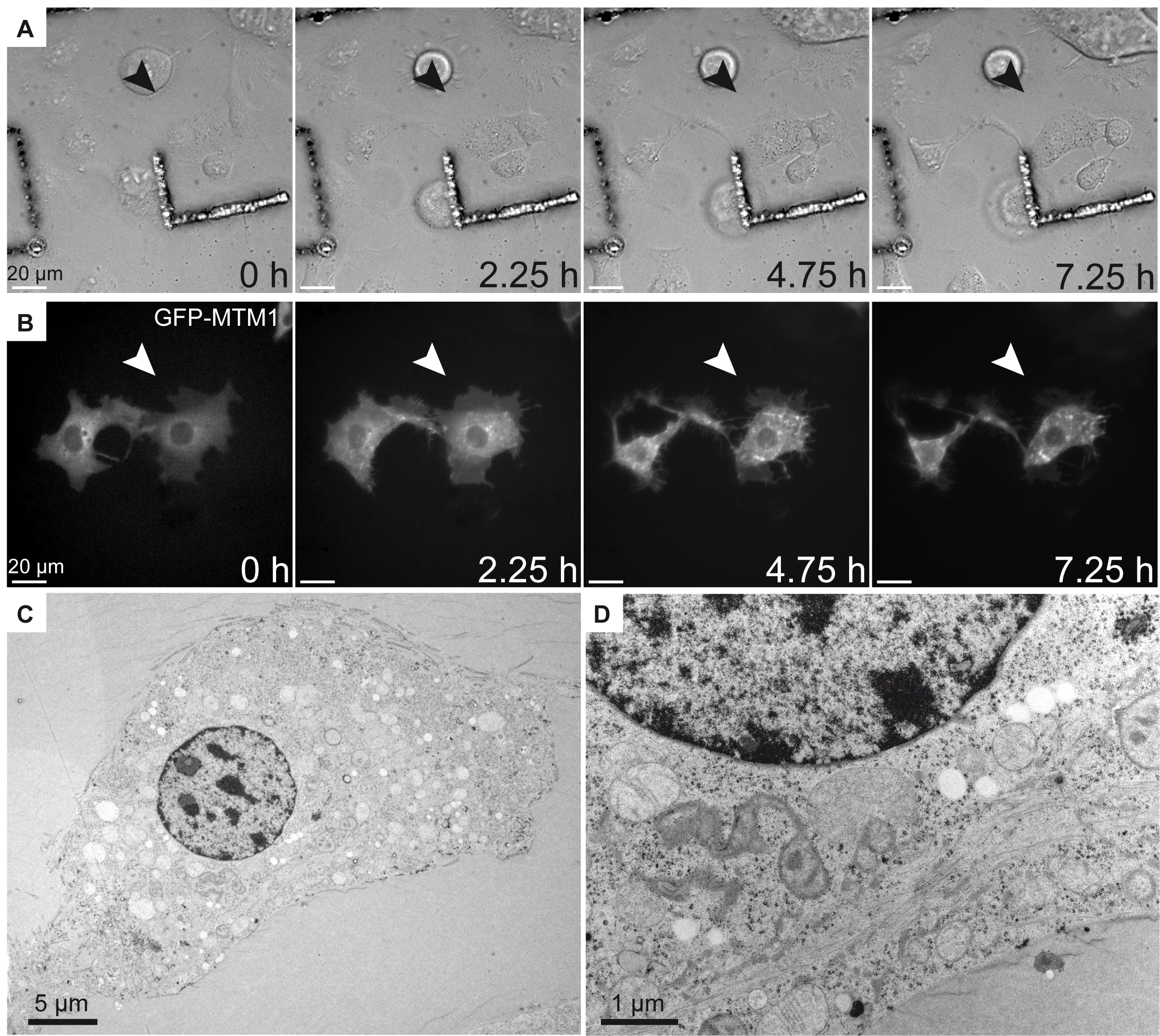

Supplement: Figure S2 — Correlative light-electron microscopy on migrating cells. Migration of COS-1 cells expressing GFP-MTM1 on the pre-patterned aclar grid coated with collagen. (A) Brightfield timelapse acquisition. (B) Corresponding fluorescence images; the arrow indicates the cell of interest. (C) A representative EM image of the cell pointed out in (A) and (B). (D) A high magnification image on a different section from the cell in (C). (6.38 MB TIF) [file pone.0009014.s002.tif]

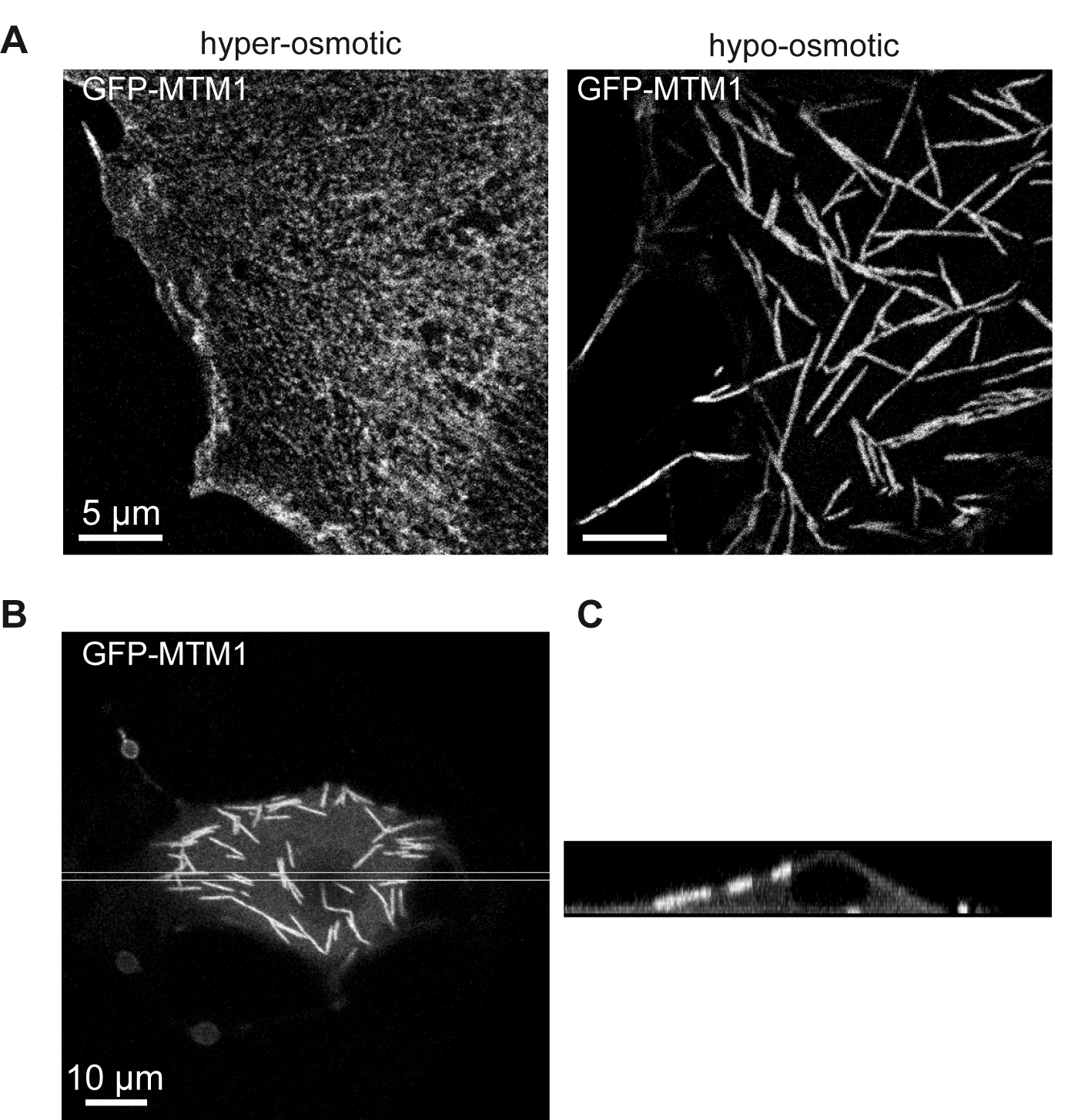

Supplement: Figure S3 — Details of the needle-like structures formed by GFP-MTM1 upon hypo-osmotic treatment. COS-1 cells were transfected with GFP-tagged MTM1 and treated for 10 min with hyper-osmotic or hypo-osmotic conditions. Protein localization was similar under normal or hyper-osmotic media. (A) xy confocal images showing the needle structures within the cell. (B) z projection image of the cell apex showing the needle organization. (C) xz projection from the image shown in (B) suggesting the presence of the needle structures at the plasma membrane and not inside the cytosol. (1.44 MB TIF) [file pone.0009014.s003.tif]

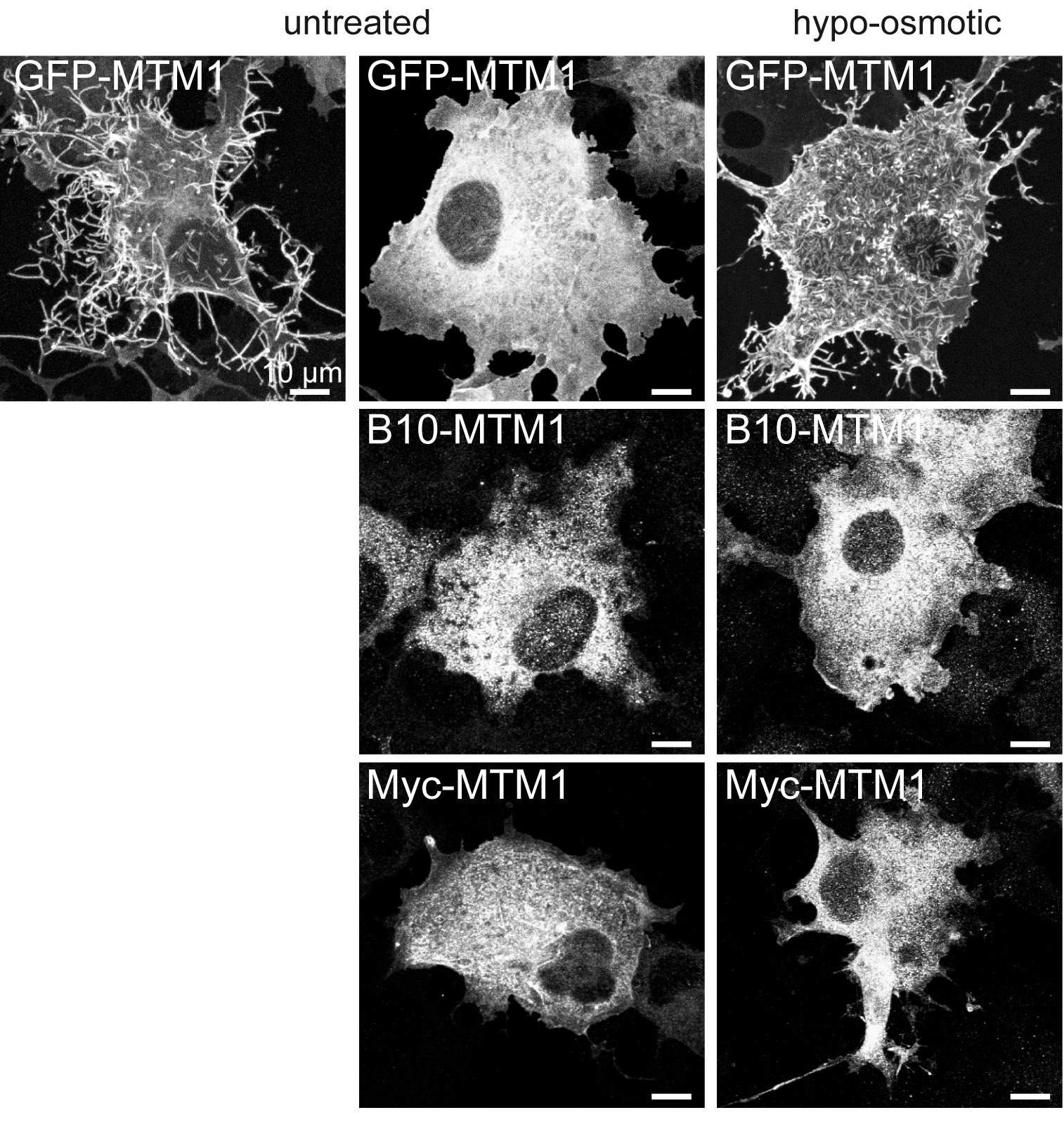

Supplement: Figure S4 — The formation of needle-like structures by MTM1 is enhanced by a GFP-tag. COS-1 cells were transfected with N-terminal B10, Myc or GFP-tagged MTM1, either untreated or switched to a hypo-osmotic medium for 10 min, and imaged by confocal microscopy. Upper left image depicts the localization of MTM1 at cell protrusions in highly over-expressing cells, while low over-expressing cells display a more diffuse cytosolic pattern. Most of B10- and Myc-tagged MTM1 transfected cells display a cytosolic pattern although some contained needle-like structures. (2.56 MB TIF) [file pone.0009014.s004.tif]

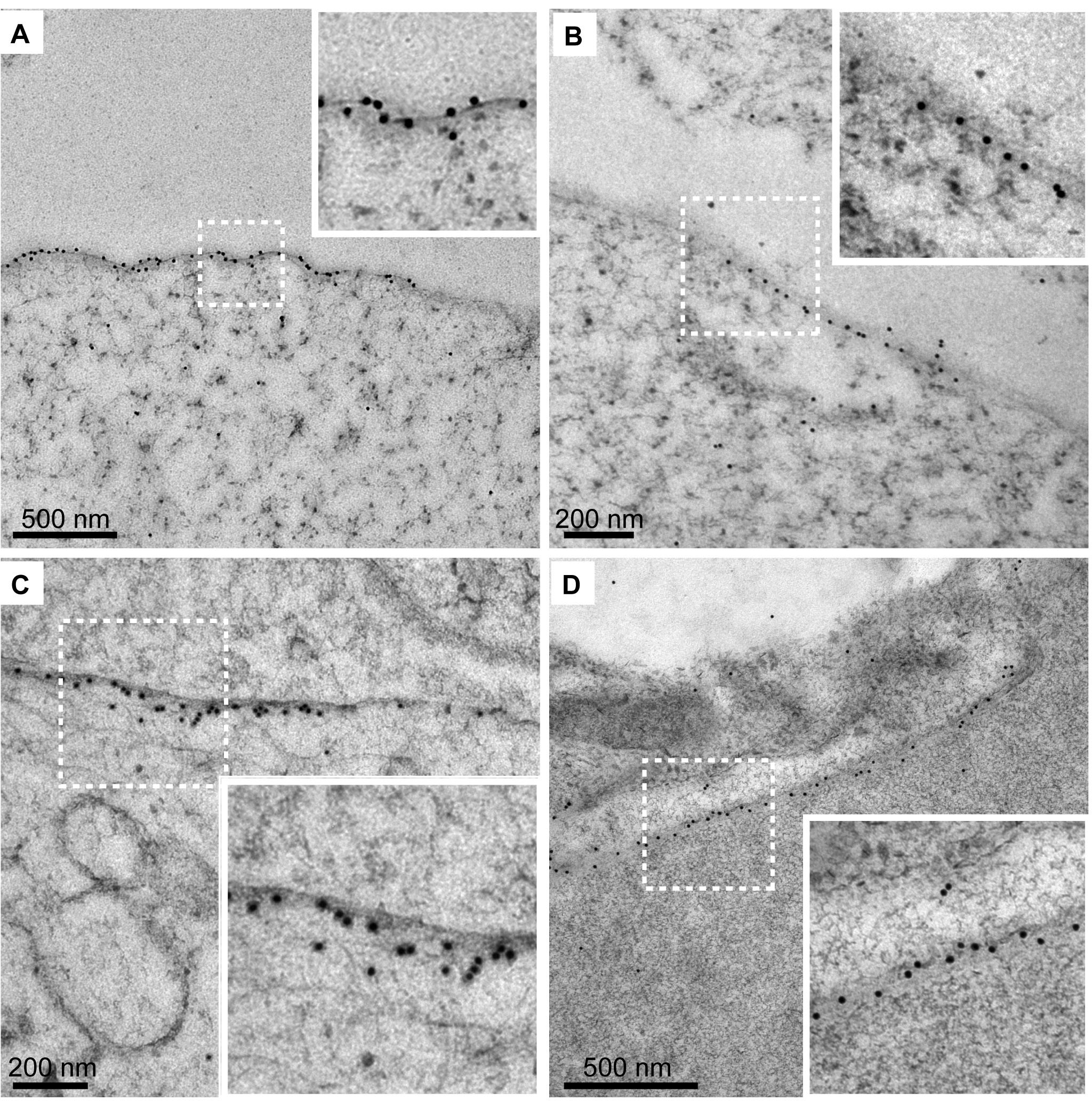

Supplement: Figure S5 — Additional GFP-positive needles observed on TEM sections after immuno-EM experiments, related to Figure 3. The gold particles, revealing the accumulation of GFP-MTM1 proteins, are concentrated at sub-domains of the plasma membrane. The inserts show magnified views of the boxed areas. (5.07 MB TIF) [file pone.0009014.s005.tif]

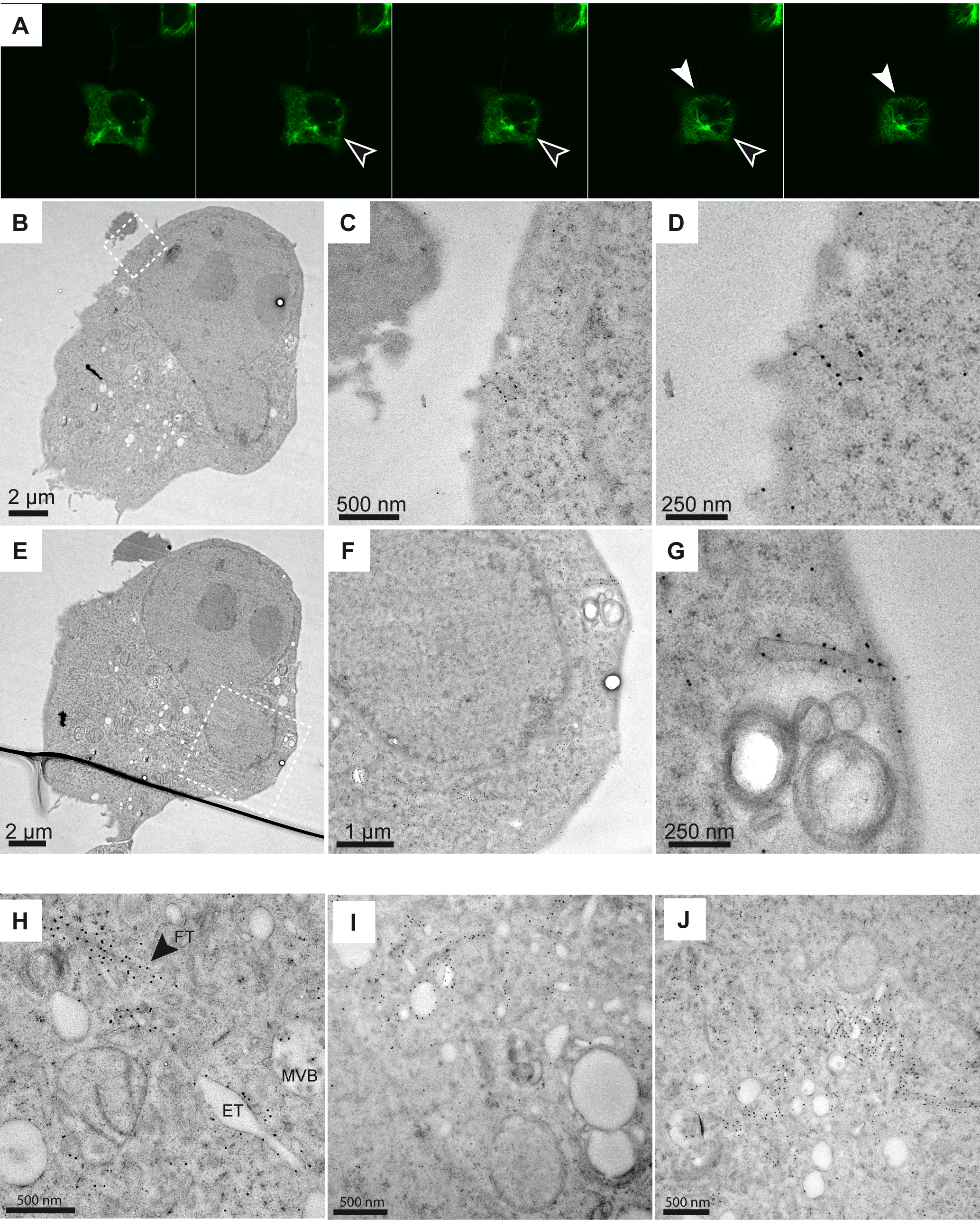

Supplement: Figure S6 — BIN1-positive structures observed after CLEM and immuno-EM. (A) Consecutive z stacks (0.28 µm thick) of the apex of the cell shown in figure 5. The white arrowheads show the region of the cell corresponding to the EM pictures shown in B-C-D and in figure 5L. The empty arrowheads point to the region where the pictures shown in E-F-G and in figure 5M were taken. (H) gold particles were associated to fine tubules (FT) and to the membrane of enlarged tubules (ET). Internal vesicles of a multivesicular body (MVB) were also stained. (I) Gold labeling was also found of vesicles of various sizes and (J) on more complex and reticulated membrane structures. These structures seem to be induced by the over-expression of the BIN1 construct as that do not appear in non transfected cells. Their identity, i.e. endosomal or lysosomal, post-golgi, is not known. (6.55 MB TIF) [file pone.0009014.s006.tif]
